# Automated Waitlists for Ambulatory Appointment Scheduling: Multisite, Mixed Methods Evaluation

**DOI:** 10.2196/90091

**Published:** 2026-07-23

**Authors:** Elizabeth Woodcock, Chris Profeta, Molly Siegel

**Affiliations:** 1 Patient Access Collaborative, Atlanta, GA, 30306, United States, 1 404-272-2274

**Keywords:** appointments, schedules, waiting lists, hospital outpatient clinics, ambulatory care, health information technology, implementation science

## Abstract

**Background:**

Delays in access to ambulatory care are associated with adverse health outcomes, diminished patient experience, and increased system inefficiencies. US health systems have adopted automated waitlists, a technology-enabled tool that notifies patients of earlier appointment availability. Existing case reports suggest benefits. However, there is limited evidence on the efficacy of adopting, implementing, and sustaining an automated waitlist to improve access.

**Objective:**

The objective of this study was to evaluate automated waitlists to identify determinants that influence their adoption and ongoing use. Findings may inform health care organizations seeking to improve access to appointments in the ambulatory setting.

**Methods:**

A convergent, multisite, mixed methods study was conducted. Data were collected through a survey of 127 health systems, with 90 reporting data about automated waitlist usage, accompanied by a criterion-based purposive study of 10 participating health systems. Both qualitative and quantitative data were collected from the 10 participating systems. The Consolidated Framework for Implementation Research was used to report the determinants of the intervention’s performance.

**Results:**

Automated waitlists provide benefits to US health systems. High-performing health systems reported that 38.8% (IQR 36.2%‐45.7%) of appointments offered by the automated waitlist were filled. Participants reported a lower missed appointment rate (3.1%, IQR 2.5%‐4.8%) for appointments scheduled via the automated waitlist, as compared to all appointments (6.6%, IQR 4.1%‐9.9%). Flexible configuration serves as a key facilitator of adoption and maintenance. External pressures, including peer benchmarking and high patient demand, accelerated implementation. Insurance and records requirements, seasonality, care plan configuration, and digital inequities controlled which patients could benefit from the intervention. Additionally, specialty gatekeeping and clinician capacity constraints limited impact. Organizational context strongly shaped effectiveness, with cross-functional governance structures, leadership endorsement, and cultures of iteration enabling sustained use.

**Conclusions:**

Automated waitlists may offer a solution to health care organizations striving to positively impact access to care. The effectiveness of automated waitlists depends primarily on the implementation process and inner-setting organizational determinants. Rather than functioning as a stand-alone technical solution, automated waitlists are most impactful when integrated as a dynamic component of system-level scheduling infrastructure.

## Introduction

**Figure 1.**
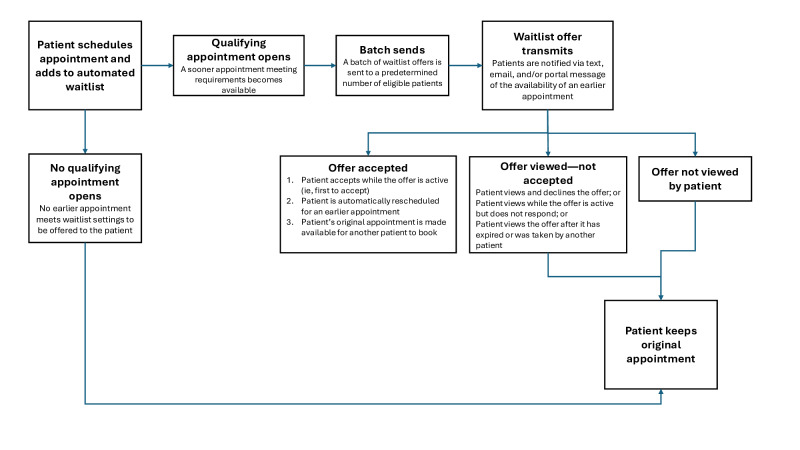
Workflow for automated waitlist.

Delays in accessing care may adversely impact health outcomes [1-5]. Patient expectations for convenient and timely access to care continue to rise [6-9], creating strain as demand surges due to delayed care during the COVID-19 pandemic and the presence of ongoing clinician workforce shortages [10-12]. Lengthy wait times are reported for ambulatory appointments [13,14]. Innovative queuing tools have been introduced in health systems to reduce operating room scheduling delays, demonstrating financial value and operational efficiency [15-17]. Automated waitlists are used in other industries to manage customer queues [18,19]. With emerging application in the ambulatory setting, automated waitlists may offer a promising approach to reducing delays to care. An automated waitlist is a technology-enabled process through which waitlisted customers receive automated notifications (eg, text, email, or message) when earlier appointment times become available. The system continuously monitors openings and sends offers without employee intervention. Customers—patients, in the case of health care organizations—may choose to accept the offered slot or, without action, retain their originally scheduled appointment in the ambulatory setting (Figure 1). Through an automated waitlist, patients may be offered a more timely appointment slot, thereby improving quality [20], safety [1-5,21], and patient experience [22-26].

For the health system, the loss of a perishable asset is avoided based on patients arriving in slots that would otherwise go unused had it not been for the waitlist, without the necessity of manual labor. Furthermore, the original appointment may be released for future consumption, thereby helping another patient due to the availability of the newly opened appointment. To date, automated waitlists have been studied within a single health system [27-30]. This study aims to fill the gap in the multisite evaluation of this intervention to improve the generalizability of findings [31-33].

Despite the need for access improvements and evidence to support the use of automated waitlists, there is an absence of literature about the intervention. To address this gap in knowledge, this study aimed to identify elements of an automated waitlist. These include evidence of access improvement and factors shaping the adoption, use, and sustainment of automated waitlists. The research findings may benefit health care organizations considering an automated waitlist as a schedule management tool to improve access to care in the ambulatory setting.

## Methods

### Overview

Relevant scholarly literature was identified and analyzed to provide the evidentiary foundation for this study. The research study used a convergent mixed methods design, integrating interviews and questionnaires to address the research objective. Data were gathered in parallel, with quantitative and qualitative components analyzed independently before being merged. The quantitative results support broader generalization, while the qualitative insights provide contextual depth by capturing participant perspectives [34]. Through the use of a mixed methods research design, the study provides more comprehensive meaning and insight [35-38] to an intervention designed to address a complex management issue in health care [39]. Generalizability may be improved based on the ability to triangulate findings furthered by the study design [40,41].

Interviews followed a semistructured interview protocol as informed by the Consolidated Framework for Implementation Research (CFIR) [42] to describe internal and external factors regarding the novel technology. Internal factors include the motivation to address a problem (eg, unused appointment slots); external factors include matching technology used by peer health systems.

The researchers achieved saturation in the interviews. The study used purposive sampling for information-rich interviews. By collecting targeted, rich data through purposive sampling [43,44] and open-ended questions [45], fewer interviews were necessary [46-49].

The qualitative study was important to understand the perception of internal stakeholders at health care organizations who are directly engaged in deploying the tool. The qualitative and quantitative data from health systems were collected concurrently, and the results were integrated. Methodological triangulation was achieved by comparing and integrating quantitative operational data with qualitative findings from the semistructured interviews to enhance understanding of the tool’s performance and improve the validity of findings [50-52].

### Participants

Participants were recruited through the Patient Access Collaborative (PAC) between September and November 2025 (qualitative). The PAC consists of 127 geographically diverse US health systems that are academic medical centers, children’s hospitals, and cancer centers. Each year, the administrators of the collaborative learning health care system [53] issue a comprehensive survey of management activities related to patient access in the ambulatory setting of its member health systems. Based on member interest, data are collected about ambulatory management solutions, including the automated waitlist. Ninety of the 127 health systems reported data in the 2025 survey regarding automated waitlist usage. Criterion-based purposeful sampling was used for the follow-up qualitative and quantitative evaluation [54].

Participants were identified based on their submission to the PAC’s annual benchmarking study. The inclusion criterion was the top 10 highest reported automated waitlist slot fill rates. The researchers sent an email communication to the main contact within the PAC’s membership database seeking participation in the study. The main contacts are senior health system leaders engaged in ambulatory access management at their health system. They were asked to identify a manager who was a primary internal stakeholder for the automated waitlists at their respective health system. Two of the 10 health systems declined to participate and an additional one cited an error in the original data submission, which removed them from the top 10. Therefore, the next 3 health systems on the list (original numbers 11‐13) were invited to participate. Ten accepted the invitation to participate. Table 1 displays the geographical distribution of the 10 criterion-based survey participants.

For the quantitative survey, 2 datasets were analyzed. First, the dataset of 90 health systems that was used to identify the top performers was analyzed. These data were collected in March and April 2025 as a component of the PAC’s annual benchmarking study. Table 2 displays the geographical distribution of the quantitative survey participants in the large dataset.

Second, the 10 participating health systems selected based on the highest automated waitlist fill rate were sent a survey in November and December 2025 (see Multimedia Appendix 1 for the criterion-based quantitative survey instrument). All surveys were completed during this time period.

**Table 1.** Patient Access Collaborative member characteristics of the 10 criterion-based survey participants.

Location	Health systems, n (%)
Midwest	1 (10)
Northeast	1 (10)
South	5 (50)
West	3 (30)

**Table 2.** Patient Access Collaborative member characteristics of health systems reporting waitlist usage for the annual benchmarking survey (N=90).

Location	Health systems, n (%)
Midwest	22 (24.4)
Northeast	21 (23.3)
South	29 (32.2)
West	18 (20)

### Data Collection

Data were collected for the quantitative and qualitative studies. For the quantitative evaluation, the annual benchmarking survey results were reviewed. The following 3 questions were asked of US health systems that are members of the PAC:

1. What is the average number of patients offered a slot opportunity per batch? (A “batch” is defined as a simultaneous distribution of waitlist appointment offers to a cohort of eligible patients.)
2. What is the average number of batches per day (as defined by a 24-hour period) distributed to patients?
3. What is the percentage of slots offered by the automated waitlist filled to a scheduled appointment? (Upon distribution of a batch, a cohort of eligible patients is simultaneously presented with an offer for an earlier appointment. Acceptance of the offer by any patient results in the automatic scheduling and fulfillment of the appointment via the waitlist, without manual intervention by the health system. An accepted offer is therefore classified as a “filled to a scheduled appointment.”)

The third question was used as the inclusion criterion to identify the 10 participants for the follow-up qualitative and quantitative study. All three questions were analyzed and reported for the full dataset.

For the criterion-based participants, the authors developed a semistructured interview guide for the qualitative interview. The survey included questions about the timing, volume, and distribution of waitlist offers, and the facilitators and barriers regarding the deployment and ongoing use of the automated waitlist.

The interview guide was tested with 2 experienced administrators. Interviews were conducted virtually between November and December 2025 using the Zoom platform and were recorded with participants’ permission. The interviews lasted 30 to 45 minutes and were transcribed verbatim. Either the author or coauthor led the interview. A minimum of 2 authors were present for all 10 interviews. The survey questions are presented in Multimedia Appendix 1.

Concurrently, the authors developed a quantitative survey instrument to gather insight about the process settings, search parameters, scheduling rules, communication techniques, and performance metrics of automated waitlists from the 10 participating US health systems. In August 2025, the survey instrument was tested with a group of 15 capacity management directors from PAC member health systems. These directors have extensive experience with the adoption and deployment of the automated waitlist. Revisions were made based on feedback from this group. For the quantitative survey, an electronic survey was distributed using the SurveyMonkey platform. The survey was transmitted to the participant upon scheduling their interview in November and December 2025. A reminder was sent if results were not received within a week of the interview. Surveys were completed in November and December 2025. The survey questions are presented in Multimedia Appendix 1.

Variables used in the analysis included lag time, missed appointment rate, and days of improvement. Lag time was defined as the number of calendar days between the patient’s appointment request date and the scheduled appointment date. The missed appointment rate was defined as the proportion of scheduled appointments for which the patient neither arrived nor provided advance notice of cancelation. Days of improvement were defined as the number of calendar days by which a patient’s appointment was moved earlier through the automated waitlist.

### Data Analysis

For the quantitative research, data from the PAC benchmarking survey were analyzed in Excel. A total of 127 health systems were surveyed about the deployment of an automated waitlist. Among responding health systems, 90 (70.9%) reported using an automated waitlist.

For the criterion-based sample, participants from 10 health systems were sent the survey upon agreeing to the qualitative interview. Ten participants completed the survey, representing a 100% response rate. The data were extracted from the survey instrument and analyzed in Excel. Descriptive statistics are reported.

For the qualitative research, transcripts were coded in NVivo (Lumivero). The researchers iteratively developed a codebook. Transcripts were analyzed using thematic analysis framed by implementation determinants and contextual factors. The CFIR domains and constructs served as the basis for the codes [42]. Thematic analysis is a qualitative method used for identifying, analyzing, and documenting patterns within data [55-57].

The analysis incorporated 5 stages [58]:

1. Familiarization involved reading all transcripts, reviewing field notes, and developing a deep understanding of the data (EW, CP, and MS).
2. Initial codes and categories were generated (MS), and a thematic index was developed, reviewed, and refined in collaboration with all authors (EW, CP, and MS).
3. All transcripts were coded using the agreed-upon index (MS).
4. Codes were charted to consolidate all data belonging to each category in a single location (MS and EW).
5. The charts were reviewed and interpreted to assess the range and strength of emerging themes, as well as the relationships among them, by all authors (EW, CP, and MS).

The coding framework was structured and reported using the CFIR [42]. This framework served as a guiding model, offering a systematic, evidence-informed approach for identifying, categorizing, and conveying the factors that shape the use of a technological intervention, thereby enhancing the potential for applying the study’s results. CFIR provides a structured yet flexible framework ideal for understanding the barriers and facilitators within complex internal and external environments such as health care organizations [59].

### Ethical Considerations

This study was reviewed by Solutions Institutional Review Board (protocol number 1092) and deemed exempt. Informed consent was obtained from all participants prior to their participation in the study. Participants did not receive compensation for their participation.

## Results

### Overview

Ninety of 127 (70.9%) health systems reported data regarding their automated waitlist tool, inferring that the tool is being used by a majority of US health systems. The median percentage of slots offered by the automated waitlist that were filled to a scheduled appointment was 25% (IQR 15%‐34%). The median number of batches per day was 3.0 (IQR 2.0‐5.0). The median number of slot opportunities per batch was 10.0 (IQR 5.0‐15.0).

Myriad process settings and scheduling rules were reported by the top 10 performing health systems. Among the top 10 participants, there was consensus on having the same appointment slot being available to schedule manually during the offer phase, the automated waitlist respecting provider session limits when generating offers, patients receiving offers for the automated waitlist via text, and patients being able to opt out of offers. Other settings and rules varied by participant. Overall, the results revealed a propensity toward the inclusion of all patients scheduled in the future. The majority of participants (8/10, 80%) included all slots available through autosearch, in addition to same-day (n=6, 60%) and next-day slots (n=9, 90%). Thirty percent (n=3) reported excluding appointments that may have insurance restrictions, while the remaining 70% (n=7) included them in the automated waitlist offers. However, 50% (n=5) of participants excluded appointments that required prior authorization. Most participants (n=8, 80%) excluded linked appointments. Participants (n=10, 100%) reported compliance with existing scheduling rules. For example, most participants respected provider session limits (n=9, 90%) and designated private (n=7, 70%), held (n=8, 80%), and overbook slots (n=7, 70%). Only 30% (n=3) of participants reported that patients were required to opt in. Table 3 provides an overview of reported settings and rules.

**Table 3.** Automated waitlist settings and rules (N=10).

Question	Yes, n (%)
Do automated waitlist offers expire once the next batch process runs?	6 (60)
If an automated waitlist offer is active, can the same appointment slot still be scheduled manually by employees?	10 (100)
Are all appointment slots identified through an autosearch process eligible to be offered via the automated waitlist?	8 (80)
Does your automated waitlist process include same-day appointments in its search parameters?	6 (60)
Does your automated waitlist process include next-day appointments in its search parameters?	9 (90)
Does your automated waitlist process respect provider session limits when generating offers?	9 (90)
Are any specialties excluded from participation in the automated waitlist process?	8 (80)
Does your automated waitlist process respect designated scheduling blocks when generating offers?	10 (100)
Are designated private slots excluded from the pool of slots offered through the automated waitlist?	7 (70)
Are designated held slots excluded from the pool of slots offered through the automated waitlist?	8 (80)
Are designated overbook slots excluded from the pool of slots offered through the automated waitlist?	7 (70)
Are patients with incomplete or missing registration data excluded from the pool of slots offered through the automated waitlist?	2 (20)
Are patients with two or more linked appointments (eg, physician appointment and imaging study) excluded from the pool of slots offered through the automated waitlist?	8 (80)
Are there restrictions on which patients can receive waitlist offers based on insurance type or coverage status?	3 (30)
Are visits identified as requiring prior authorization excluded from the pool of slots offered through the automated waitlist?	5 (50)
Does your automated waitlist strategy differ between new and established patients?	6 (60)
Are patient cohorts prioritized for receiving waitlist offers (eg, by clinical acuity, new vs established status, employees, recent cancelations)?	5 (50)
Are patients required to opt in to receiving automated waitlist offers?	3 (30)
May patients opt out of receiving automated waitlist offers?	10 (100)
Do patients receive and respond to automated waitlist offers via text message?	10 (100)
Do patients receive and respond to automated waitlist offers via email?	9 (90)
Do patients receive and respond to automated waitlist offers via patient portal?	9 (90)

According to the high-performing health systems, the median frequency of batches executed was 4.5 (IQR 4.0‐5.8), higher than the 3.0 (IQR 2.0‐5.0) reported by all health systems. The maximum number of patients who can be offered each available appointment slot per batch process run by the automated waitlist was 10.0 (IQR 10.0‐10.0). The most cited scheduled time at which the automated batch process runs was 6 PM, as reported by 60% (6/10) of respondents. Fifty percent (n=5) of respondents reported sending a batch at 5 PM, followed by 40% (n=4) at 7 AM, 9 AM, 4 PM, 6 PM, and 7 PM; 30% (n=3) at 8 AM and 5 PM; 20% (n=2) at 6 AM, 10 AM, 1 PM, and 2 PM; and 10% (n=1) at 9 PM. The look-ahead period, at minimum, was 0.17 (IQR 0.17‐1.75) calendar days (4.1 h) with a maximum of 30.0 (IQR 16.0‐30.0) calendar days.

The median number of offers each patient could receive per day was 1.0 (IQR 1.0‐2.0). Seven of the 10 (70%) health systems set their minimum improvement in appointment timing required for a patient to receive a waitlist offer at 3 days. For the top 10 performing health systems, 14.4% (IQR 7.6%‐34.8%) of automated waitlist offers were accepted by patients. A total of 38.8% (IQR 36.2%‐45.7%) of appointments offered by the automated waitlist were filled, higher than the 25.0% (IQR 15.5%‐33.5%) automated waitlist slot fill rate reported by all health systems. Thirty-six (IQR 30.0‐44.0) days of improvement were realized. Accepted waitlist offers brought the patient’s appointment dates forward by 36 (IQR 30.0‐44.0) calendar days from the original appointments, thus shortening the lag time for the patients. There were 8 (IQR 6.3‐24.1) calendar days between the offer acceptance date and the offered appointment date. Accepted waitlist appointments were scheduled 8 (IQR 6.3‐24.1) calendar days from the date of offer acceptance. The originally scheduled appointments were 44 days in the future, whereas the accepted waitlist appointments were 8 days in the future, yielding a reduction in lag time of 36 calendar days. Participants reported a lower missed appointment rate (3.1%, IQR 2.5%‐4.8%) for appointments scheduled via the automated waitlist, as compared to all appointments (6.6%, IQR 4.1%‐9.9%). Table 4 presents parameters reported by the top 10 performing health systems.

**Table 4.** Automated waitlist parameters (N=10).

Parameter	Respondents, median (IQR)
Frequency of the automated waitlist batch process executed each day, as defined by a 24-h period	4.5 (4.0‐5.8)
Maximum number of automated waitlist offers a patient can receive in a single day	1.0 (1.0‐2.0)
Maximum number of patients who can be offered each available appointment slot per batch process run by the automated waitlist	10.0 (10.0‐10.0)
Minimum look-ahead period (in calendar days) used to identify available open slots for the automated waitlist process	0.17 (0.17‐1.75)
Maximum look-ahead period (in calendar days) used to identify available open slots for the automated waitlist process	30.0 (16.0‐30.0)
Minimum improvement in appointment timing (in calendar days) required for a patient to receive a waitlist offer	3.0 (3.0‐3.0)
Waitlist acceptance rate, %	14.4 (7.6‐34.8)
Waitlist slot fill rate, %	38.8 (36.2‐45.7)
Median number of days by which patients’ appointments are advanced (“days of improvement”) as a result of the waitlist process	36.0 (30.0‐44.0)
Median number of lag days (in calendar days) between the offer acceptance date and the offered appointment date	8.0 (6.3‐24.1)

The qualitative survey results highlighted the importance of themes in the integration and decisions surrounding automated waitlists. The responses were evaluated and coded according to the CFIR constructs. Twenty-two unique codes were available to the researchers. The researchers determined that the constructs of teaming and doing within the implementation process would be combined, as well as engaging and adapting.

Of the 295 total mentions, the implementation process was discussed most, with 127 (43.1%) mentions, followed by the inner setting with 77 (26.1%) mentions, innovation with 56 (18.9%) mentions, individuals with 24 (8.1%) mentions, and the outer setting with 11 (3.7%) mentions.

**Figure 2.**
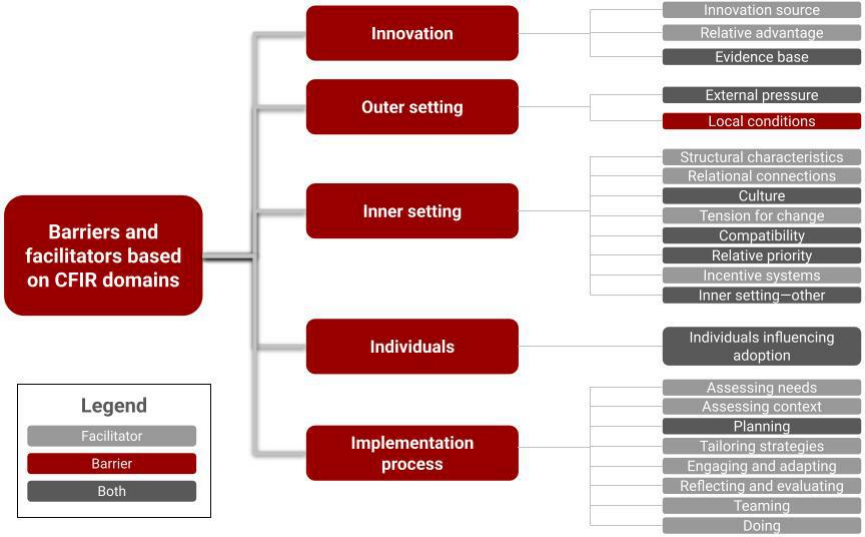
Automated waitlist barriers and facilitators. CFIR: Consolidated Framework for Implementation Research.

Figure 2 highlights the facilitators and barriers identified in the qualitative interview process based on the CFIR domains.

### Domain 1: Innovation

Across the participating US health systems, automated waitlists were widely perceived as an innovation with a clear relative advantage over manual workflows:

*There’s no one left to sit on the phone calling patients to ask if they want to come sooner. [The automated waitlist] does what we simply can’t do anymore*.

Further, the prevention of nonarrival presented a quantifiable relative advantage:

*[W]aits were so long that patients were getting seen somewhere else. When [the automated waitlist] started filling those earlier slots, we saw no-shows drop*.

Early pilots served as strong facilitators, particularly in specialties with lengthy appointment wait times. As a participant explained:

*When we tried it in our pilot department, it worked really well, and that early success made everyone else want to get on board*.

Flexibility in configuration also supported implementation, with organizations adjusting geolocation rules, insurance authorization requirements, message frequency, and visit-type eligibility. Another participant reported:

*We tinkered a lot with the batches and timing, and that adaptability is what helped us get to 85 percent utilization*.

However, several intervention-specific constraints posed barriers. The inability to move linked or sequential appointments together limited adoption in specialties requiring tightly coordinated care. A participant described:

*If a patient has multiple appointments that need to stay together, the system just does not know how to keep them linked*.

Additionally, some clinical areas viewed early movement as unsafe due to care plan timing, such as gestational-stage requirements or mandated record review periods.

### Domain 2: Outer Setting

External pressures and patient needs strongly influenced implementation. Peer benchmarking motivated adoption, with 1 participant describing how external comparison created *“a huge push, because everyone else seemed to be using a waitlist and talking about their results.*” High patient demand was also a key facilitator. “We were shocked by how many patients were willing to travel or switch locations just to get in sooner,” a participant observed.

Barriers in the outer setting stemmed from insurance rules. Some organizations required prior authorization or financial clearance before offers could be sent, which restricted who could receive earlier appointment options. As a participant stated:

*You [referring to a patient] have to be ‘cleared’ before the offer can go out, and that alone knocks out a big group of patients*.

These requirements were a function of the health system, not the technology. Seasonality further shaped performance, with lower provider availability during holidays reducing offer volume.

### Domain 3: Inner Setting

The inner setting emerged as one of the strongest determinants of effectiveness. Governance structures, including access committees, dashboards, and cross-functional working groups, facilitated alignment across operations, information technology, patient experience, and clinical leadership. An interviewee conveyed:

*We had operations, IT, training, and clinical leadership all at the table. That governance structure was what allowed the tool to work and continue working*.

A participant described this as a “roadshow of communication,” noting that leadership “gave us moments to showcase the work at every major forum.”

A culture of piloting and iteration also supported success. “We always start with, let’s pilot it and see what happens,” a participant reported. “If it fails, we regroup and try again.” Incentive structures tied to access performance metrics reinforced adoption and created shared accountability among internal stakeholders.

Conversely, specialty-based gatekeeping frequently constrained tool use. “Some departments said they didn’t want to use it because they preferred to control access themselves,” a participant explained. Certain workflows were deeply manual or highly customized, making integration challenging. Capacity constraints such as clinician shortages also limited the number of earlier appointments available to patients.

### Domain 4: Individuals

Individual attitudes and engagement shaped implementation variability across sites. Champions within specialties experiencing high demand drove early momentum. “They were the group that said, we’ll try anything,” a participant noted, describing a department that adopted the tool eagerly and demonstrated strong early results. Executive leadership endorsement further strengthened adoption:

*Our CEO gave us time in major meetings to highlight the wins, and that made a big difference*.

However, skepticism and clinical concerns among some clinicians acted as barriers. Some clinicians worried that the tool might “overbook patients” or disrupt established workflows. Others were hesitant due to perceived risks of moving patients earlier in the care pathway. Individual comfort with automation varied widely, creating inconsistent uptake across specialties.

### Domain 5: Implementation Process

The implementation of the automated waitlist required continual assessment, tailoring, and adaptation. Stakeholders repeatedly refined offer batches, notification timing, parameters, and communication content. As a participant summarized:

*It is never a set-it-and-forget-it process. We are always tweaking something*.

Patient engagement efforts, such as directly asking patients about their preferred reminder frequencies, strengthened message tailoring and acceptability.

Ongoing monitoring facilitated improvement. Health systems built custom dashboards to monitor acceptance rates, lag reduction, and refill rates. A participant noted:

*We created our own reporting because the canned reports did not make sense. We needed data we could trust*.

An indicator of the efficacy of the automated waitlist, the refill rate is defined as the proportion of available appointment slots offered via the waitlist that result in a successfully scheduled appointment. This metric is distinct from the offer acceptance rate, for which the denominator comprises all offers distributed to patients. Given that a single available appointment may be extended to multiple patients, the refill rate evaluates the effectiveness of the waitlist in filling open capacity on the appointment schedule, whereas the acceptance rate reflects the volume of offers required to achieve a single acceptance.

Premature or poorly sequenced implementation created challenges:

*When we first turned [the automated waitlist] on, it crashed and burned. We hadn’t done the education or the buy-in. When we came back with a phased, intentional rollout, it was a completely different experience*.

A participant shared an instance of negative patient feedback tied to message timing, prompting a major rework:

*One complaint made us rethink the entire communication cadence*.

The concern for the experience of patients was cited by interviewees; for example:

*[W]e learned that sending more offers gets more fills, but it also means more patients get rejected. It’s a balance between efficiency and experience*.

Operational constraints, such as the need for record review or pending insurance authorization, required careful planning to avoid inappropriate offers. Consideration regarding the potential for inequity was raised:

*Everything is done through text, so whoever sees it first gets the spot. Some patients never even have a chance*.

Another participant provided insight into access to patients who may not receive the communication, revealing a potential structural inequity:

*We only offer [the automated waitlist] in English and Spanish. If a patient needs Creole, for example, they won’t get an offer. The technology just isn’t built for that yet*.

## Discussion

### Overview

To the best of our knowledge, this is the first multisite study of automated waitlists in the ambulatory setting of the US health care system. Data collection was achieved using a survey of 127 health systems, with 90 reporting data about automated waitlist usage, accompanied by a criterion-based purposive study of 10 participating health systems. Qualitative and quantitative data were collected from the 10 participants. Through an assessment of the quantitative and qualitative data, automated waitlists may positively impact access to care.

This study examined the implementation of automated waitlists as a schedule management tool within the ambulatory setting. Schedule management is considered an important internal effort for health system administrators to address growing appointment lead times [60,61]. Automated waitlists may operate as an integral component of a schedule management strategy, helping organizations proactively fill appointment openings and reduce wait times.

The effectiveness of automated waitlists is shaped less by the technology itself than by how the intervention is configured, embedded, and governed within the health care organization. Across sites, automated waitlists functioned not as a static tool but as adaptive infrastructure, requiring ongoing alignment with ambulatory workflows, operational priorities, and patients’ needs.

Although automated waitlists are widely used in other industries, their adoption in health care depends on their adaptability to heterogeneous clinical environments. Participants described tailoring eligibility rules to account for clinical appropriateness, insurance authorization requirements, and patient behavior. These adaptations reflect a broader principle observed in implementation science: outcomes depend on how technologies are operationalized within local contexts rather than on their technical capabilities in isolation [62]. Table 5 lists the adaptability factors used by participants.

**Table 5.** Automated waitlist adaptability factors.

Element of automated waitlist	Example of adaptation
Insurance coverage and requirements	Prior authorization or network status for the upcoming appointment
Medical record review	Availability of or time to review the medical record prior to the upcoming appointment
Selection of priority populations	Health system employees
Geographical or site preference	Reasonable mileage to the upcoming appointment or preferred location from the patient’s residence
Linked appointments	Upcoming appointments incorporating multiple encounters such as physician appointments and imaging studies
Volume of offers per patient	Number of notifications transmitted to each patient
Patient agreement	Opt in or opt out
Visit type	New vs established patient
Clinical parameters	Existence of a care plan
Appointment horizon	Days or hours prior to the upcoming appointment
Communication method	Text, email, portal message, or other
Access opportunity	Current performance related to new patient access, cancelation rate, etc
Patient behavior	Patients’ historical reaction to waitlist offers
Patient characteristics	Age, gender, and primary language of the patient
Clinician adoption readiness	Acceptance of self-service tools and waitlist-driven scheduling
Slot availability	Existing constraints (or lack thereof) to appointment bookings
Maturity of clinicians’ practice	New clinicians working with clinicians with mature patient panels
Specialty/subspecialty of clinician	Clinical specialty
Offer expiration	Minutes until the waitlist offer expires for the upcoming appointment
Patient access to tool	Portal uptake, broadband access, availability of cellular minutes, language, and literacy

By contrast, manual waitlists were described as costly to maintain, difficult to scale, and often ineffective without dedicated oversight [63,64]. Automated waitlists offer a clear advantage over manual ones, providing capabilities that cannot be matched in terms of efficiency, consistency, or scale.

The research findings highlight important equity considerations associated with automated waitlists. By systematically offering earlier appointments to patients experiencing long waits, the intervention reduced reliance on chance, patient persistence, or informal appointment navigation strategies.

There is evidence of a digital divide among patients [65-67]. The digital divide refers to the unequal distribution of access to digital technology among individuals. Appointment offers made available by an automated waitlist require timely engagement with a digital tool. Therefore, patients with greater access to digital devices, broadband, and flexible schedules were more likely to benefit. Participants noted limitations in language-concordant communication, which excluded some patients from participation altogether.

These findings suggest that automated waitlists may simultaneously mitigate and reproduce structural inequities. Equity must be intentionally incorporated into the design and deployment determinations of an automated waitlist. These efforts may include raising awareness about equity [68], engaging patients with lived experience in the tool’s implementation strategy [69], and integrating considerations for the digital divide into the tool’s workflow [70]. Organizational context emerged as a critical determinant of success. Implementation results depended on alignment across operational, technical, and clinical stakeholders, supported by governance structures such as committees or workgroups with shared accountability.

Effective governance of technology is a vital enabler of safety, value realization, strategic alignment, and sustainability in health care organizations by structuring processes, roles, decision rights, and accountability in implementation and use [71,72]. The findings reinforce prior evidence that organizational culture, particularly norms around trust, flexibility, and shared ownership [73,74], plays a more decisive role in the impact of automated waitlists than the innovation itself. In settings where the culture was characterized by control or gatekeeping, the tool’s effectiveness was markedly constrained. Where leaders visibly endorsed the automated waitlist and created space for shared learning, adoption accelerated and performance improved.

Notably, the role of individual champions was limited. The absence of reliance on specific individuals reflects the system-level orientation of automated waitlists. Effective implementation of technology aimed at addressing a system-level opportunity in a complex, adaptive health care organization is shaped by the interplay and mutual influence of determinants [75,76]. Outcomes are formulated by the interaction of myriad organizational-level factors rather than individual agency.

The implementation process itself was also consequential. Participants described early failures when automated waitlists were deployed without adequate education, stakeholder engagement, or phased rollout.

Intentional implementation strategies that emphasized communication, incremental activation, and iterative adjustment yielded more favorable results. Identical technologies offered different results depending on the local context and implementation strategy, rather than the features of the tool alone [77-80].

Organizations also navigated trade-offs between operational efficiency and patient experience; increasing the volume of appointment offers improved fill rates but led to more patient rejections, requiring careful calibration.

The balance of patient experience and operational effectiveness is critical to achieving a successful outcome. Assessing needs, tailoring strategies, and engaging with patients are vital for health care organizations using an automated waitlist.

Automated waitlists may serve as an important component of a schedule management strategy rather than as a stand-alone optimization tool. When thoughtfully implemented, the technology may assist health care organizations in proactively managing capacity, reducing wait times, and improving access to care in the ambulatory setting. Without deliberate attention to equity, governance, and implementation strategy, automated waitlists risk amplifying existing disparities or underperforming relative to their potential.

Future research should examine equity impacts more directly and explore design approaches that expand the benefits of automation to patients historically underserved by digital access solutions.

### Limitations

The criterion-based purposive sample of 10 participants consisted of a small sample. Automated waitlists represent an emerging technology, which allowed saturation with a small sample because of nonprobabilistic sampling [49] and salience [81]. Access to care represents a multifactorial problem [82-86]. The research study did not seek to prove a causal link between the automated waitlist and improved access to care. Through the tool, a patient is presented with an opportunity to move up in time to an earlier appointment. It may be assumed that the original appointment may be available for use by another patient; however, the authors did not examine the outcome of the original appointment. The results are presented to inform health care organizations of a potential tool that may influence access to care. The participants in the surveys represent academic health systems, children’s hospitals, and cancer centers in the United States; the ambulatory enterprises associated with these health systems are large and complex, thereby limiting the generalizability of the results. The cost of the automated waitlist was not addressed with interview participants. The research study did not contain the opinions of suppliers (persons creating the technology) or patients (persons using the technology). This omission may result in an incomplete understanding of the technology use, as the viewpoints of internal stakeholders administering the technology may not fully capture the experiences of suppliers or patients [87]. The lack of standard naming conventions may have resulted in missing or errant data. An agreement on common factors to identify and monitor the impact of automated waitlists is recommended. The automated waitlist is not currently designed to account for acuity; this warrants interest for future research.

### Conclusion

An examination of the characteristics, benefits, and usage of automated waitlists provides insight for health care organizations contemplating the implementation or expansion of this consumer-facing digital tool. The implementation determinants and contextual influences related to automated waitlist adoption and performance may inform stakeholders. An automated waitlist may offer a solution to health systems striving to positively affect access to ambulatory care.

## Supplementary material

10.2196/90091 Multimedia Appendix 1 Qualitative and quantitative survey instruments for the criterion-based interviews.

10.2196/90091 Checklist 1 A checklist for mixed methods research manuscript preparation and review.

## Abbreviations

CFIR: Consolidated Framework for Implementation Research
PAC: Patient Access Collaborative
