# Supplementary material for: Automated Waitlists for Ambulatory Appointment Scheduling: Multisite, Mixed Methods Evaluation
Source: J Med Internet Res. 2026 Jul 23;28:e90091. doi: 10.2196/90091 (PMC13395261; doi:10.2196/90091)
Supplement: Multimedia Appendix 1 [file jmir-v28-e90091-s001.docx]

*Qualitative Survey Instrument*. 1. What data or evidence supported the health system’s decision to adopt this automated waitlist tool? 2. How would you describe your health system’s readiness to adopt and sustain automated waitlist? 3. How do patient preferences or feedback influence the configuration or use of the automated waitlist? 4. Are there payer requirements, quality measures, or regulatory standards that affect how your automated waitlist operates? 5. To what extent do comparisons with peer institutions or industry benchmarks influence your waitlist strategy or performance goals? 6. How is the performance or effectiveness of the automated waitlist process communicated internally (e.g., to staff, managers, and clinicians)? 7. What factors determine the utilization of your automated waitlist functionality by internal stakeholders? (Please describe key facilitators and barriers.) 8. What approaches have been effective in maintaining clinician engagement for the automated waitlist? (Please describe strategies, incentives, or communication methods used.) 9. How are the language, communication method, and message content for patient-facing waitlist offers determined? (Please describe the process or criteria used.) 10. Are the accepted waitlist offer outcomes included in other patient access metrics (e.g., new patient lag, self-scheduling rate, etc.) 11. Are there any success stories that you could share with us?

*Quantitative Survey Instrument*.

Approximately how frequently is your automated waitlist batch process executed each day, as defined by a 24-hour period? For example, the batches are executed 3 times per 24-hour period.

Please indicate the scheduled times at which the automated waitlist batch process runs.

What is the maximum number of automated waitlist offers a patient can receive in a single day? (Please provide a numeric response, e.g., 2.)

What is the maximum number of patients who can be offered each available appointment slot per batch process run by the automated waitlist? (Please provide a numeric response, e.g., 10.)

Do automated waitlist offers expire once the next batch process runs?

If an automated waitlist offer is active, can the same appointment slot still be scheduled manually by staff?

Are all appointment slots identified through the automated search (i.e., autosearch) process eligible to be offered via the automated waitlist?

Does your automated waitlist process include same-day appointments in its search parameters?

Does your automated waitlist process include next-day appointments in its search parameters?

What is the minimum look-ahead period (in calendar days) used to identify available open slots for the automated waitlist process? (Example: 1 day prior to opening)

What is the maximum look-ahead period (in days) used to identify available open slots for the automated waitlist process? (Example: 150 days prior to opening)

What is the minimum improvement in appointment timing required for a patient to receive a waitlist offer?

Does your waitlist process respect the following when generating offers? (Provider Session Limits, Scheduling Blocks, Other)

Are these categories excluded in the pool of slots offered through the automated waitlist?

- Private Slots
- Held Slots
- Overbook Slots
- Patients with incomplete or missing registration data
- Patients with two or more linked appointments
- Visit(s) that are identified as requiring prior authorization

If there are other categories that are excluded from the pool of offered slots, please describe:

Are there restrictions on which patients can receive waitlist offers based on insurance type or coverage status?

Are any specialties excluded from participation in the waitlist process?

Which specialties have demonstrated the most substantial performance gains (e.g., increased slot utilization, reduced lag time, or improved fill rates) attributable to the automated waitlist? (Please list up to five specialties.)

Does your automated waitlist strategy differ between new and established patients? If yes, please describe. E.g., differences in logic, eligibility, or prioritization.

Are patient cohorts prioritized for receiving waitlist offers (e.g., by clinical acuity, new vs. established status, employees, recent cancellations)?

Are patients required to opt in to receiving automated waitlist offers?

May patients opt out of receiving waitlist offers?

Through which modalities can patients receive and respond to waitlist offers? (Select all that apply.)

What is your current waitlist acceptance rate (i.e., the percentage of waitlist offers that are accepted by patients)? (Please provide a percentage value.)

What is your waitlist slot fill rate (i.e., the percentage of appointment openings successfully filled through the waitlist process)? (Please provide a percentage value.)

What is your late-cancellation refill rate (i.e., the percentage of late cancellations successfully filled via the waitlist)? (Please provide a percentage value.)

How do you define a late cancellation?

What is the average number of days by which patients’ appointments are advanced (“days improved”) as a result of the waitlist process? (Please provide a numeric value.)

What is the average number of lag days between the offer acceptance date and the offered appointment date?

What is your overall no-show rate across all scheduled appointments? (Please provide a percentage value.)

What is your no-show rate for appointments scheduled through the automated waitlist process? (Please provide a percentage value.)
