# Supplementary material for: Automated Waitlists for Ambulatory Appointment Scheduling: Multisite, Mixed Methods Evaluation
Source: J Med Internet Res. 2026 Jul 23;28:e90091. doi: 10.2196/90091 (PMC13395261; doi:10.2196/90091)
Supplement: Checklist 1 [file jmir-v28-e90091-s002.docx]

**Multimedia Appendix. A Checklist for Mixed Methods Research (MMR) Manuscript Preparation and Review.**

*Lee SD, Iott B, Banaszak-Holl J, Shih SF, Raj M, Johnson KE, Kiessling K, Moore-Petinak N. Application of Mixed Methods in Health Services Management Research: A Systematic Review. Med Care Res Rev. 2022;79(3):331-344. PMID:34253078*

Rational and description of MMR design

Provide a clear statement of the study purpose

*To date, automated waitlists have been studied within a single health system* [1–4]*. This study aims to fill the gap in multisite evaluation of this intervention to improve the generalizability of findings* [5–7]*.*

Explicitly describe the MMR design in accordance with Creswell’s (2015) typology and use a diagram to illustrate the relationship and sequence of qualitative and quantitative research components

Results

Compare & Contrast

Quantitative

Data collection & analysis

Results

Qualitative

Interpretation

Data collection & analysis

Justify why the MMR design is appropriate for meeting the study purpose.

*The quantitative results support broader generalization, while the qualitative insights provide contextual depth by capturing participant perspective* [8]. *Additionally, the design was appropriate for the study purpose based on the design supporting:*

- *The ability to triangulate findings to improve generalizability* [9,10]
- *A pragmatic approach to the research* [11,12]
- *An attempt to determine a more comprehensive meaning of the intervention* [13,14] *and insight about the intervention itself* [15]
- *The intervention involves an attempt to solve a complex health care management issue* [16]

Transparency in describing method details

Describe the study population(s) and sample(s; e.g., who, what, how many)

*Participants were recruited through the Patient Access Collaborative between September and November 2025 (qualitative). The Patient Access Collaborative (PAC) consists of 127 geographically-diverse US health systems that are academic medical centers, children’s hospitals, and cancer centers. Ninety of the 127 health systems reported data regarding automated waitlist usage. A criterion-based purposeful sampling was used for the follow-up qualitative and quantitative evaluation. Participants were identified based on their submission to the PAC’s annual benchmarking study.*

Describe the sampling procedures (including inclusion and exclusion criteria, recruitment)

*The inclusion criterion was the top-10 highest reported automated waitlist slot fill rates. The researchers sent an email communication to the main contact within the Patient Access Collaborative’s membership database seeking participation in the study. The main contacts are senior health system leaders engaged in ambulatory access management at their health system. They were asked to identify a manager who was a primary internal stakeholder for the automated waitlists at their respective health system. Two of the ten health systems declined to participate and an additional one cited an error in the original data submission which removed them from the top 10. Therefore, the subsequent three were invited to participate. Ten accepted the invitation to participate. Table 1 displays the geographical distribution of the 10 criterion-based survey participants.*

*Table 1. Location of Ten Criterion-Based Survey Participants*

| *Participants’ Location* | *Count* | *Percent of Total* |
| --- | --- | --- |
| *Midwest* | *1* | *10%* |
| *Northeast* | *1* | *10%* |
| *South* | *5* | *50%* |
| *West* | *3* | *30%* |

*For the quantitative survey, two data sets were analyzed. First, the data set of 90 health systems that was used to identify the top performers was analyzed. These data were collected in March and April 2025 as a component of the PAC’s annual benchmarking study. Table 2 displays the geographical distribution of the quantitative survey participants in the data set.*

*Table 2. Location of Survey Participants Reporting Waitlist Usage*

| *Participants’ Location* | *Count* | *Percent of Total* |
| --- | --- | --- |
| *Midwest* | *22* | *24.4%* |
| *Northeast* | *21* | *23.3%* |
| *South* | *29* | *32.2%* |
| *West* | *18* | *20.0%* |

*Second, the 10 participating health systems selected based on the highest automated waitlist fill rate were sent a survey in November and December 2025. (See Supplemental Material for Criterion-based Quantitative Survey Instrument.) All surveys were completed during this time period.*

Describe qualitative data collection processes (how often data were collected, who collected the data, what kind of data collection instruments were used, how data were recorded—e.g., notes, transcripts)

*Data were collected for the qualitative studies. For the criterion-based participants, the authors developed a semi-structured interview guide for the qualitative interview. The interview guide was tested with two experienced administrators. Interviews were conducted virtually between November and December 2025 using the Zoom® technology platform and were recorded with participant permission. The interviews lasted 30 to 45 minutes and were transcribed verbatim. Either the author or co-author led the interview. A minimum of two authors were present for all ten interviews. The survey questions are presented in the multimedia appendix.*

Describe quantitative data collection processes (how often data were collected, who collected the data, what kind of data collection instruments were used measurements, validity/reliability)

*Data were collected for the quantitative studies. For the quantitative evaluation, the annual benchmarking survey results were reviewed. Three questions were asked of US health systems that are members of the Patient Access Collaborative:*

*1. What is the average number of patients offered a slot opportunity per batch?*

*2. What is the average number of batches per day (as defined by a 24-hour period) distributed to patients?*

*3. What is the percentage of slots offered by the automated waitlist filled to a scheduled appointment?*

*The third question was used as the inclusion criteria to identify the 10 participants for the follow- up qualitative and quantitative study. All three questions were analyzed and reported for the full data set.*

*The authors developed a quantitative survey instrument to gather insight about the process settings, search parameters, scheduling rules, communication techniques, and performance metrics of automated waitlists from the 10 participating US health systems. In August 2025, the survey instrument was tested with a group of 15 capacity management directors from Patient Access Collaborative member health systems. These directors have extensive experience with the adoption and deployment of the automated waitlist. Revisions were made based on the feedback from this group. For the quantitative survey, an electronic survey was distributed using the Survey Monkey® technology platform. The survey was transmitted to the participant upon scheduling their interview in November and December 2025.*

*A reminder was sent if results were not received within a week of the interview. Surveys were completed in November and December 2025. The survey questions are presented in the multimedia appendix.*

Describe qualitative data analysis processes (coding, single or multiple coders, replication logic, credibility)

*For the qualitative research, transcripts were coded in NVivo®. The researchers iteratively developed a codebook. Transcripts were analyzed using themes related to implementation determinants and contextual factors. Thematic analysis is a qualitative method used for identifying, analyzing, and documenting patterns within data* [17–19]*.*

*The analysis incorporated five stages* [20]*:*

*1. Familiarization involved reading all transcripts, reviewing field notes, and developing a deep understanding of the data.*

*2. Initial codes and categories were generated, and a thematic index was developed, reviewed, and refined in collaboration with all authors.*

*3. All transcripts were coded using the agreed-upon index.*

*4. Codes were charted to consolidate all data belonging to each category in a single location.*

*5. The charts were reviewed and interpreted to assess the range and strength of emerging themes, as well as the relationships among them.*

*The coding framework was structured and reported using the Consolidated Framework for Implementation Research (CFIR)* [21]*. This framework served as a guiding model, offering a systematic, evidence-informed approach for identifying, categorizing, and conveying the factors that shape the use of a technological intervention, thereby enhancing the potential for applying the study’s results. CFIR provides a structured yet flexible framework ideal for understanding the barriers and facilitators within complex internal and external environments like health care organizations.*

Describe quantitative data analysis procedures (missing data and how they are handled, statistical tests used)

*For the quantitative research, data from the PAC benchmarking survey were analyzed in Excel®. 127 health systems were asked to report on their automated waitlist; 90 participated, representing a 70.9% response rate….For the criterion-based sample, participants from 10 health systems were sent the survey upon agreeing to the qualitative interview. Ten participated, representing a 100% response rate. The data were extracted from the survey instrument and analyzed in Excel®.* *Descriptive statistics are reported.*

Integration of qualitative and quantitative research components.

*Methodological triangulation was achieved by comparing and integrating quantitative operational data with qualitative findings from the semi-structured interviews to enhance understanding of the tool’s performance and improve the validity of findings* [22–24]*.*

Interpret qualitative analysis results with appropriate quotes if necessary.

The qualitative analysis is reported in the Discussion section with appropriate quotes.

Interpret quantitative analysis results in consideration of statistical significance, selection bias, and threats to validity

The quantitative analysis is reported in the Discussion section. Bias and threats to validity are addressed in the Limitations section.

Compare qualitative and quantitative results

*The quantitative results support broader generalization, while the qualitative insights provide contextual depth by capturing participant perspectives. Through the use of a mixed methods research design, the study provides more comprehensive meaning and insight* [11,13,15,25] *to an intervention designed to address a complex management issue in health care* [16]*.*

Address divergencies and inconsistencies between qualitative and quantitative results

*Generalizability may be improved based on the ability to triangulate findings furthered by the study design* [9,10]*.*

References:

1. North F, Buss RJ, Nelson EM, Thompson MC, Pecina J, Miller NE, Crum BA. Enhancing the performance of patient appointment scheduling: outcomes of an automated waitlist process to improve patient wait times for appointments. Health Serv Insights. 2025;18:11786329251326461. doi: 10.1177/11786329251326461
2. Chung S, Martinez MC, Frosch DL, Jones VG, Chan AS. Patient-centric scheduling with the implementation of health information technology to improve the patient experience and access to care: retrospective case-control analysis. J Med Internet Res. 2020;22(6):e16451. doi: 10.2196/16451
3. Yin ALL, Feigelman AM, Delgado Y, Min RJ, Cheriff AD, Travis Gossey J, Trepp R, Beecy A. Implementing epic fast pass for echocardiogram and endoscopy to improve healthcare access and utilization. npj Health Syst. 2024;1(1):3. doi: 10.1038/s44401-024-00005-0
4. Ganeshan S, Liu AW, Kroeger A, Anand P, Seefeldt R, Regner A, Vaughn D, Odisho AY, Mourad M. An electronic health record–based automated self-rescheduling tool to improve patient access: retrospective cohort study. J Med Internet Res. 2024;26:e52071. doi: 10.2196/52071
5. Gold JL, Dewa CS. Institutional review boards and multisite studies in health services research: is there a better way? Health Serv Res. 2005;40(1):291–308. doi:10.1111/j.1475-6773.2005.00354.x
6. Green LW, Glasgow RE. Evaluating the relevance, generalization, and applicability of research: issues in external validation and translation methodology. Eval Health Prof. 2006;29(1):126–153. doi: 10.1177/0163278705284445
7. Øvretveit J, Leviton L, Parry G. Increasing the generalisability of improvement research with an improvement replication programme. BMJ Qual Saf. 2011;20 Suppl 1(Suppl_1):i87-91. doi: 10.1136/bmjqs.2010.046342
8. Johnson RB, Onwuegbuzie AJ. Mixed methods research: a research paradigm whose time has come. Educ Res. 2004;33(7):14–26. doi: 10.3102/0013189X033007014
9. Castro FG, Kellison JG, Boyd SJ, Kopak A. A methodology for conducting integrative mixed methods research and data analyses. J Mix Methods Res. 2010;4(4):342–360. doi: 10.1177/1558689810382916
10. Hadi MA, Alldred DP, Closs SJ, Briggs M. Mixed-methods research in pharmacy practice: basics and beyond (part 1). Int J Pharm Pract. 2013;21(5):341–345. doi: 10.1111/ijpp.12010
11. Creswell JW, Clark VLP. Designing and Conducting Mixed Methods Research. 2nd edition. Thousand Oaks, CA: SAGE Publications; 2011. ISBN:978-1-4129-7517-9
12. Ridde V, Olivier de Sardan J-P. A mixed methods contribution to the study of health public policies: complementarities and difficulties. BMC Health Serv Res. 2015;15(Suppl 3):S7. doi: https://doi.org/10.1186/1472-6963-15-s3-s7
13. McKim CA. The value of mixed methods research: a mixed methods study. J Mix Methods Res. 2017;11(2):202–222. doi: 10.1177/1558689815607096
14. Creswell JW, Clark VLP. Designing and conducting mixed methods research. 3rd edition. Thousand Oaks, CA: SAGE Publications; 2017. ISBN:978-1-4833-4698-4
15. Creswell JW, Clark VLP. Designing and Conducting Mixed Methods Research. Thousand Oaks, CA: SAGE Publications; 2007. ISBN:978-1-4129-2792-5
16. Guetterman TC, Fetters MD, Creswell JW. Integrating quantitative and qualitative results in health science mixed methods research through joint displays. Ann Fam Med. 2015;13(6):554–561. doi: 10.1370/afm.1865
17. Scharf J, Vu-Eickmann P, Angerer P, Müller A, In der Schmitten J, Loerbroks A. Work-related intervention needs of medical assistants and how to potentially address them according to supervising general practitioners: a qualitative study. Int J Environ Res Public Health. 2022;19(3):1359. doi: 10.3390/ijerph19031359
18. Höppchen I, Ullrich C, Wensing M, Poß-Doering R, Suda AJ. Safety culture in orthopedics and trauma surgery : a qualitative study of the physicians’ perspective. Unfallchirurg. 2021;124(6):481–488. doi: 10.1007/s00113-020-00917-0
19. Braun V, Clarke V. Using thematic analysis in psychology. Qual Res Psychol. 2006;3(2):77–101. doi: 10.1191/1478088706qp063oa
20. Pope C, Ziebland S, Mays N. Qualitative research in health care. Analysing qualitative data. BMJ. 2000;320(7227):114–116. doi: 10.1136/bmj.320.7227.114
21. Damschroder LJ, Reardon CM, Widerquist MAO, Lowery J. The updated Consolidated Framework for Implementation Research based on user feedback. Implement Sci. 2022;17(1):75. doi: 10.1186/s13012-022-01245-0
22. Morse JM. Approaches to qualitative-quantitative methodological triangulation. Nurs Res. 1991;40(2):120–123. PMID:2003072
23. Denzin NK. The research act: a theoretical introduction to sociological methods. New York: Routledge; 2017. ISBN:978-0-202-36248-9
24. Bekhet AK, Zauszniewski JA. Methodological triangulation: an approach to understanding data. Nurse Res. 2012;20(2):40–43. doi: 10.7748/nr2012.11.20.2.40.c9442
25. Creswell JW, Hirose M. Mixed methods and survey research in family medicine and community health. Fam Med Community Health. 2019;7(2):e000086. doi: 10.1136/fmch-2018-000086
